# Using X-Ray Mammograms to Assist in Microwave Breast Image Interpretation

**DOI:** 10.1155/2012/235380

**Published:** 2012-03-22

**Authors:** Charlotte Curtis, Richard Frayne, Elise Fear

**Affiliations:** ^1^Department of Electrical and Computer, Schulich School of Engineering, University of Calgary, Calgary, AB, Canada T2N 1N4; ^2^Department of Radiology, University of Calgary, Calgary, AB, Canada T2N 2T9

## Abstract

Current clinical breast imaging modalities include ultrasound, magnetic resonance (MR) imaging, and the ubiquitous X-ray mammography. Microwave imaging, which takes advantage of differing electromagnetic properties to obtain image contrast, shows potential as a complementary imaging technique. As an emerging modality, interpretation of 3D microwave images poses a significant challenge. MR images are often used to assist in this task, and X-ray mammograms are readily available. However, X-ray mammograms provide 2D images of a breast under compression, resulting in significant geometric distortion. This paper presents a method to estimate the 3D shape of the breast and locations of regions of interest from standard clinical mammograms. The technique was developed using MR images as the reference 3D shape with the future intention of using microwave images. Twelve breast shapes were estimated and compared to ground truth MR images, resulting in a skin surface estimation accurate to within an average Euclidean distance of 10 mm. The 3D locations of regions of interest were estimated to be within the same clinical area of the breast as corresponding regions seen on MR imaging. These results encourage investigation into the use of mammography as a source of information to assist with microwave image interpretation as well as validation of microwave imaging techniques.

## 1. Introduction

X-ray mammography is the current gold standard breast imaging technique [1]. Mammography provides high-resolution 2D images of the breast in each of the cranial-caudal (CC) and medial-lateral oblique (MLO) directions and is capable of resolving fine structures such as microcalcifications. However, mammography has been shown to have low sensitivity and specificity among premenopausal women and women with dense breasts [2]. In cases where mammography is ambiguous, complementary imaging techniques such as magnetic resonance (MR) or ultrasound may be used.

Different modalities rely on different tissue properties in order to generate an image; for example, X-ray image contrast results from tissue density, whereas ultrasound imaging relies on acoustic impedance. Examining multiple modalities can provide diagnostic information that might be missed if only a single imaging technique was used [2]. Because of the advantages of combining information from different sources, there is benefit in continuing to develop new imaging methods.

Various studies have measured differences in the electromagnetic (EM) properties of fatty, fibroglandular, and cancerous breast tissues, suggesting a possible source of image contrast [3]. These differences are the basis for development of patient-friendly, safe, and inexpensive imaging techniques. Specifically, EM tomography and radar-based approaches have been proposed [4, 5]. Both techniques require the woman to lie prone on the examination table with the breast extending through a hole in the table top into a tank of immersion liquid; this is similar to patient positioning in MR image acquisition, with the additional presence of the immersion liquid [6]. An antenna emitting low-powered nonionizing EM radiation is then used to illuminate the breast. The waves travelling through the breast are reflected from internal structures and recorded on one or more receiving antennas.

Tissue sensing adaptive radar (TSAR) is a 3D radar-based microwave imaging technique that is currently in the preliminary stages of clinical testing [7]. Compared to MR (the current 3D breast imaging modality), TSAR is less costly, less invasive as no contrast agent is required, and does not exclude patients with metallic implants or claustrophobia [6]. While initial results are promising, it is challenging to interpret the TSAR images without 3D images collected with another modality. To this end, the preliminary TSAR study included collection of MR images. However, MR scans are not typically part of patient care and add considerable time and expense to research studies.

Mammograms are a routine procedure in breast cancer cases [1]. Using these data to assist in interpreting TSAR would remove the dependence on MR in future studies, allowing for a greater patient cohort. However, mammograms cannot be directly compared to 3D imaging techniques. The specific aim of this work is to develop a method of interpreting mammograms in 3D. While the ultimate goal is to visualize mammograms in conjunction with TSAR images, MR images provide an effective tool to assist in the development and validation of this approach.

To use mammograms to interpret TSAR data, the 2D information must be translated into 3D space. However, mammograms are only obtained at two orientations, approximately 45° apart. Furthermore, the breast is compressed up to 50% of its original diameter, causing significant distortion of the 2D images [8]. These two issues are the main challenges in estimating 3D information from mammograms.

This paper presents a method to estimate the 3D skin surface and 3D location of regions of interest from standard two-view mammograms. The accuracy of the estimation is quantified by directly comparing to MR images and computing the difference between estimated and true skin surface in four anatomical directions.

Previous work in reconstructing the surface of the skin from mammograms has been presented in only three related publications [9–11]. The technique employed by Yam et al. and Kita et al. involved eroding the breast contour to compensate for compression, followed by fitting curves under the assumption that the MLO view approximates the ML view [9, 10]. Behrenbruch et al. refined this work by registering mammograms to MR images for more accurate compression compensation [11]. However, none quantified the accuracy of their results to known 3D geometry; this work will address that issue. Similarly, the same works present methods for estimating the 3D location of suspicious regions seen on mammograms, but due to differences in acquisition geometry only Behrenbruch et al. were able to directly compare to MR images, while Kita used relative metrics. 

## 2. Methods

**Figure 1:**
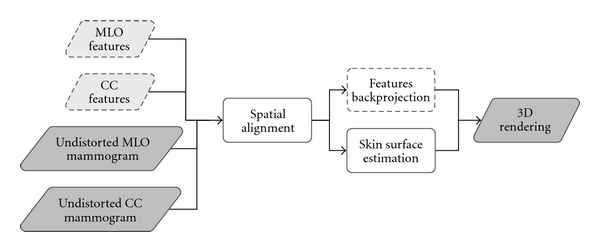
Overview of the estimation algorithm. Dotted outlines indicate steps performed only on images with identifiable features.

Figure 1 shows an overview of the methods used to estimate ROIs and the skin surface in 3D.

First, the distortion due to compression is compensated through registration with a 3D reference shape. Next, the two mammographic views and their corresponding skin contours are aligned spatially to represent physical imaging planes. The contours are then used to estimate the skin surface by fitting ellipses to coronal slices of the breast at equally spaced intervals. In cases where features such as lesions could be identified on all data sets, the 3D locations of the features are estimated through 3D backprojection. Finally, the skin surface and features are rendered in 3D and compared to MR images. 

### 2.1. Mammogram Preprocessing

In acquiring mammograms, the breast is compressed up to 50% of its original diameter, causing significant anatomical distortion of the tissues and leading to CC and MLO projection images consisting of different tissue configurations [12]. An estimation of the projections through the undistorted breast shape is therefore desirable in order to estimate the 3D structures. In this work, distortion of the mammograms was reduced through registration with projection images formed from MR images [13]; in the future, it is anticipated that TSAR images will be used to create these projection images.

**Figure 2:**
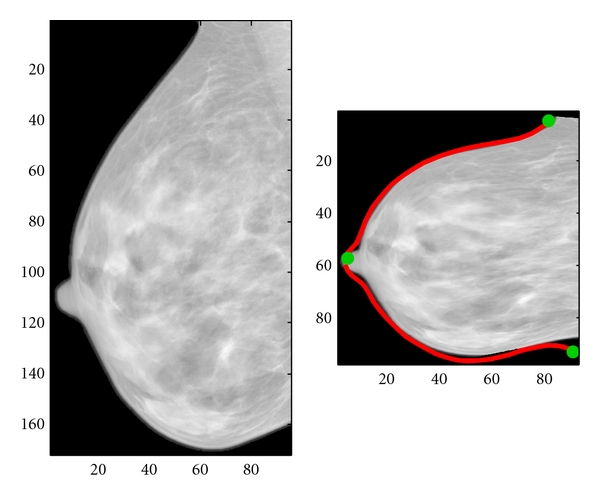
Original mammogram (left) was registered to an MR projection image to remove the distortion resulting from mammographic compression. The resulting image (right) is reduced to approximately 70% of the original surface area.

The registration technique used a combination of landmark- and intensity-based registration to reduce distortion of both the external shape and the internal tissues of the mammograms [13]. This technique is similar to that described by Behrenbruch et al. [11]. In addition to registration of the two images, a contour defining the breast boundary and the locations of three anatomical landmarks were automatically obtained through segmentation of the background followed by curvature computation.  Figure 2 shows an example of the difference in shape between the original mammogram and the undistorted version. Due to the expansion of tissues during mammographic compression, the surface area of the undistorted mammogram is approximately 30% smaller than the original.

The three circular markers of Figure 2 are anatomical landmarks located at the nipple and the two regions of maximum curvature where the breast meets the chest wall. The contour defining the boundary of the breast as seen on the MR projection is shown to illustrate the accuracy of the shape following registration. After this preprocessing step, only mammographic data are used to obtain the 3D estimates.

### 2.2. Spatial Alignment

**Figure 3:**
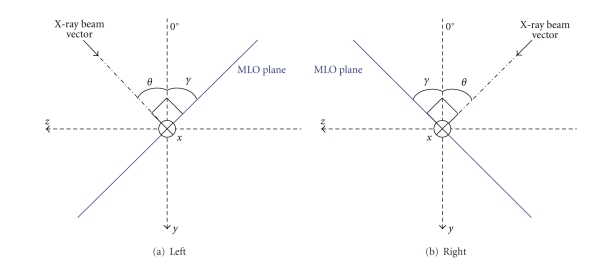
Illustration of MLO mammographic acquisition angles.

The preprocessing procedure of the previous section served to map the 2D mammographic projections into an undistorted 2D projection space. However, the relative alignment of the CC and MLO mammograms is unknown, as a given feature visible in the projected image can be located at any point along the X-ray beam vector (illustrated in Figure 3). Certain assumptions about the spatial alignment of the CC and MLO breast contours must be made in order to begin estimating the skin surface.

Unlike previous work, which assumed a 90° difference of projection angles between the CC and MLO views, this work uses the MLO acquisition angle provided in the metadata of digital mammograms. The MLO data (image, landmarks, and contour) were rotated according to the reciprocal of this angle in order to spatially align the two views. Figure 3 shows an example of a typical MLO acquisition angle *θ*, with *γ* indicating the angle of rotation applied to the MLO image and contour data. Using the coordinate system shown in Figure 3, the CC view was rotated by 90° so as to lie on the *zx* plane.

Following rotation, translation of the two images was required to further align the views in an approximation of acquisition geometry. The nipple landmarks were chosen as the [0,0, 0] spatial coordinate, as by definition the landmarks in the two images are at the same location.

Further spatial alignment required further assumptions.

- The contour of the mammogram, describing the largest edge or shadow of the breast, is located at the centre of the volume along the X-ray beam vector.
- The MLO data provide nipple location in the *y*-axis.
- The CC data provide nipple location in the *z*-axis.
- The centroid of a coronal slice taken at the chest wall corresponds to the intersection of the midpoints between the chest wall landmarks of both views.

**Figure 4:**
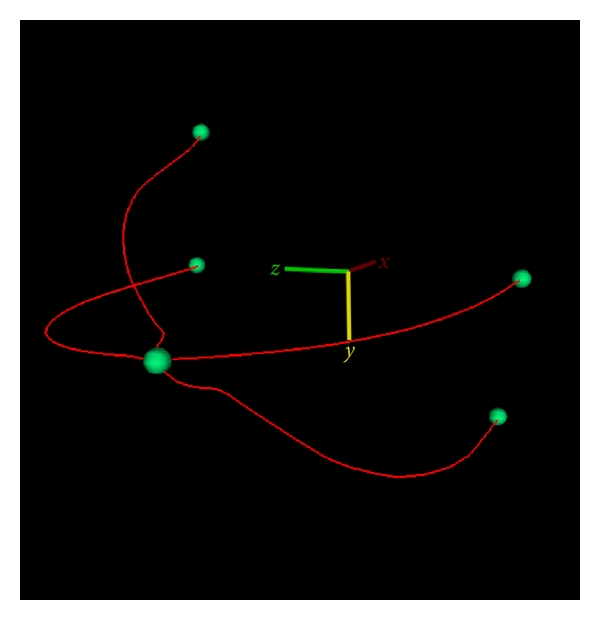
Sparse wireframe formed by spatially aligning CC and MLO breast contours.

Using these assumptions, the two image contours were aligned by rotating the CC view about the *z*-axis and the MLO view about the *y*-axis. The resulting geometry is an estimate of the orientation of the two imaging planes during acquisition of the mammograms. An example of the two contours, which form a sparse wireframe model, is shown in Figure 4. These data alone are used as the prior information for the skin surface estimation.

### 2.3. Skin Surface Estimation

**Figure 5:**
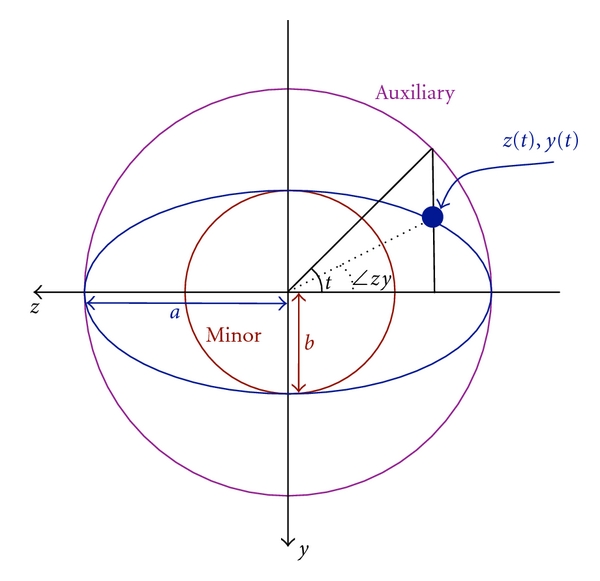
Ellipse in canonical position (*ϕ* = 0, [*z*_*c*_, *y*_*c*_] = [0,0]).

Examination of a given *zy* or coronal plane of the breast as seen in MR reveals that the breast is roughly elliptical in cross-section [14]; this fact has been used in producing breast models for numerical simulations and will be used to build the skin surface model in this work. The ellipse equation in parametric form is

(1) $$\begin{array}{cc} z\left(t\right) & =z_{c}+a\cos t\cos\phi -b\sin t\sin\phi ,\\ y\left(t\right) & =y_{c}+a\cos t\sin\phi +b\sin t\cos\phi , \end{array}$$

where [*z*_*c*_, *y*_*c*_] is the centre of the ellipse, *a* and *b* are the scaling factors for the major and minor axes, respectively, *ϕ* is the angle of the major axis, and the parameter *t* ranges from 0 to 2*π*. These variables are depicted in Figure 5.

Any coronal plane intersects the wireframe model of Figure 4 at four points. However, four points are insufficient data for ellipse fitting. Furthermore, the four intersected points are not orthogonal to each other, resulting in an uneven distribution around the ellipse.

To obtain the best fitting ellipse for the four known data points of a coronal slice, the centre point [*z*_*c*_, *y*_*c*_] was assumed to be the centroid of the four points. The rotation angle *ϕ* was set to zero, reducing (1) to two equations with three unknowns *a*, *b*, and *t*. The *t* parameter was estimated using the angle of the vector from the centre point to the absolute value of each data point (*∠* *zy* in Figure 5), providing four solutions to (1) and allowing for a least squares fit to be determined. The data were then rotated iteratively to various angles *ϕ*, and the least squares fitting was repeated until the best *ϕ* was determined.

**Figure 6:**
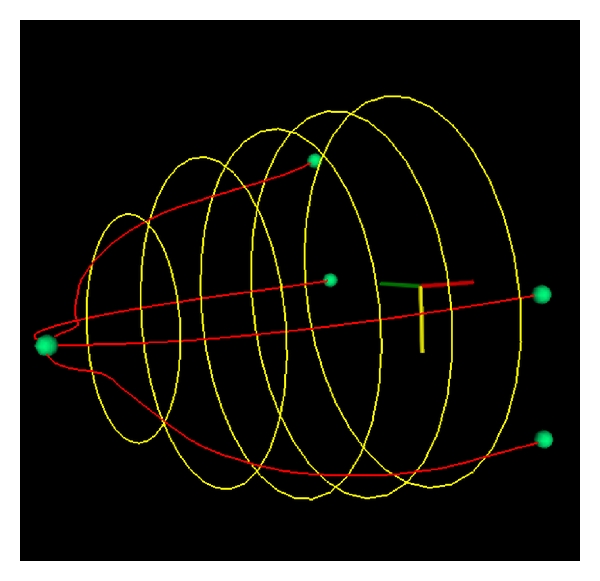
Sample skin surface estimation.

With all the parameters of (1) determined, an ellipse was created and displayed at the specified *x* location. This process was repeated for the desired number of ellipses along the *x* axis; Figure 6 shows a sample skin surface estimation. In this example, only five coronal slices are shown for clarity; in evaluating results, twenty slices will be rendered for a more complete skin surface estimation.

### 2.4. Internal Feature Estimation

The skin surface estimate described in the previous section describes the general shape of the breast and provides a frame of reference to compare the two modalities. However, identification of a particular region of interest (ROI) is of even greater utility, providing the 3D location of particular features of the breast. As mammograms provide 2D views of ROIs, these regions can be identified and their 3D location computed and displayed relative to the skin surface estimation.

**Figure 7:**
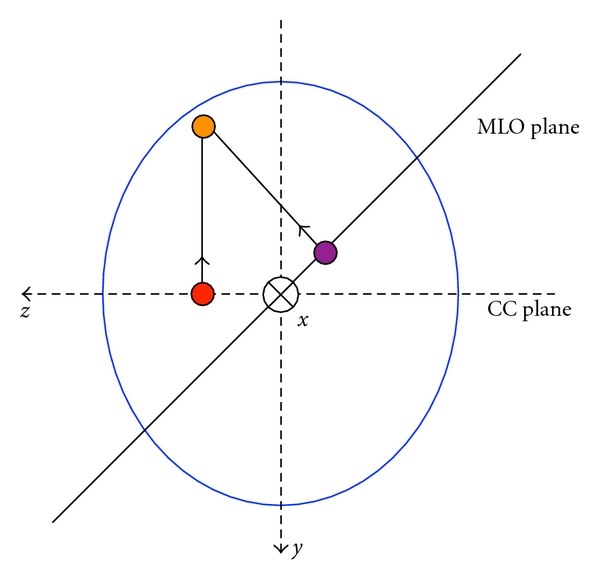
Reconstruction of internal feature points.

For the purpose of identifying the general ROI in 3D space, ROIs as seen on the undistorted mammograms were modelled as simple spheres. Corresponding features on the CC and MLO views were identified in consultation with a radiologist and marked as 2D circles through an interactive display. These points were then located in 3D space using only the mammographic data by calculating the intersection of the two lines orthogonal to the imaging planes as illustrated by Figure 7.

This is computed as follows:

(2) $$\begin{array}{cc} x & =\frac{x_{\text{cc}}+x_{\text{mlo}}}{2},\\ y & =y_{\text{mlo}}+\frac{z_{\text{mlo}}-z_{\text{cc}}}{\tan^{-1}\gamma },\;\\ z & =z_{\text{cc}}, \end{array}$$

with *γ* as indicated in Figure 3. The radius of the 3D sphere was taken as the average of the two 2D circles.

## 3. Results and Discussion

The skin surface estimation technique was applied to six sets of patient data, resulting in a total of twelve breast models. Mammograms were collected digitally according to the Canadian standards, yielding CC and MLO images at varying resolutions. Both contrast-enhanced and fat-suppressed T1-weighted MR images were collected, also in compliance with the Canadian breast imaging protocols. For the purposes of image registration and comparison with mammography, the fat-suppressed structural MR images were used.

With the assistance of a radiologist, regions of interest on the data sets were identified. Only one was a candidate for internal feature location estimation due to a need for discrete lesion visibility on all three images (CC, MLO, and MR).

For each data set, an MR image was loaded and displayed to provide ground truth geometry. This image was aligned as accurately as possible relative to the feature estimation model, but it should be noted that alignment is subject to errors due to the assumptions made in aligning the wireframe model.

### 3.1. Skin Surface Estimation

**Figure 8:**
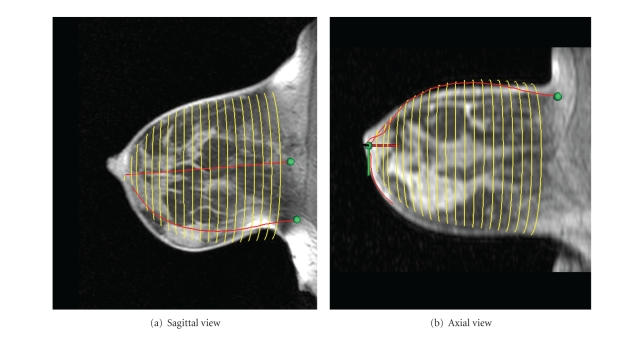
Comparison of estimated skin surface with MR (sample data set).

**Figure 9:**
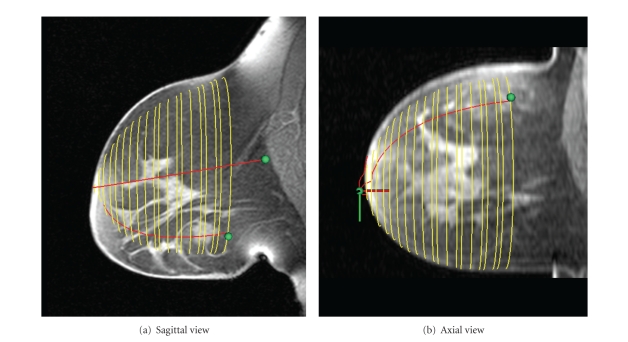
Comparison of estimated skin surface with MR (data set with lesion).

Figures 8 and 9 show sagittal and axial views of a central MR slice with the estimated skin surface overlaid.  Figure 8 is the data set used to describe methods, while Figure 9 is the data set containing the lesion.

Examination of the skin estimations of both Figures 8 and 9 shows that, despite independent reconstruction at each coronal slice, a smooth contour is achieved. Furthermore, the algorithm succeeds in capturing the breast shape even when large interpatient variations are present.

The sagittal view of the estimation shown in Figure 9(a) shows that the ellipse technique has failed to completely capture the cranial-caudal asymmetry of the breast shape. This is likely due to the large breast making contact with the side of the MR coil, resulting in a downturned nipple and distorted MR geometry. However, the axial view (undistorted at this location) shows excellent medial-lateral correspondence.

**Figure 10:**
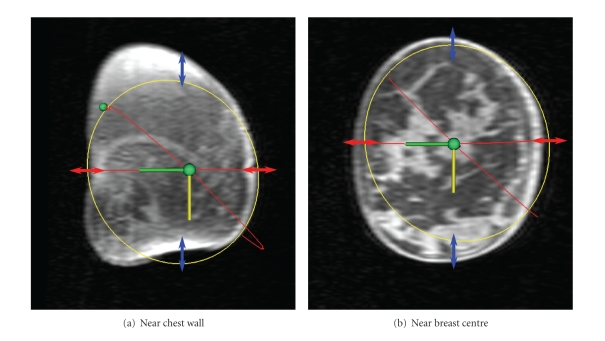
Comparison of estimated skin surface with MR. Arrows indicate error between estimated and true skin surface at four anatomical directions (arrow size exaggerated for visibility).

A coronal cross-section of the sample data set at two different locations is shown in Figure 10. Due to deformation of the breast near the chest wall caused by contact with the MR coil, the estimated ellipse fails to accurately capture the shape at this location (Figure 10(a)). However, near the centre of the breast, the ellipse approximation is more reasonable (Figure 10(b)). To quantify errors in estimated skin outline, the difference between the true skin surface as seen in a coronal slice of the MR image and the estimated surface was calculated at the medial, lateral, cranial, and caudal directions. These errors are indicated by the arrows in Figure 10.

**Figure 11:**
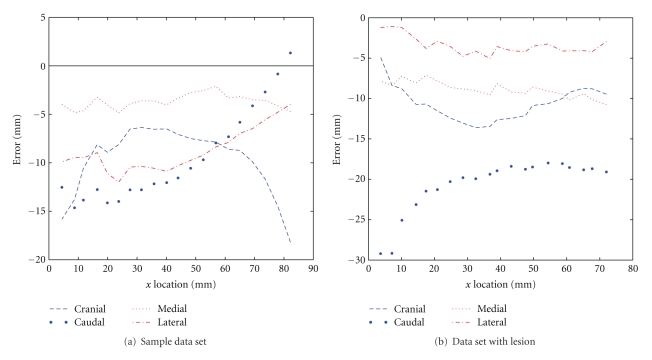
Skin estimation error versus coronal slice (*x*) location for two data sets.

The skin estimation errors were computed such that negative errors represent underestimation of the breast surface. To observe the relationship between coronal slice location and estimation error, the errors were plotted against coronal slice location (*x*-axis). The resulting error plots are shown in Figure 11 for the two sample data sets. In both cases, it is clear that the cranial and caudal errors tend to exceed the medial and lateral errors. Furthermore, all of the errors tend to be negative, suggesting a consistent underestimation of the true skin surface.

**Figure 12:**
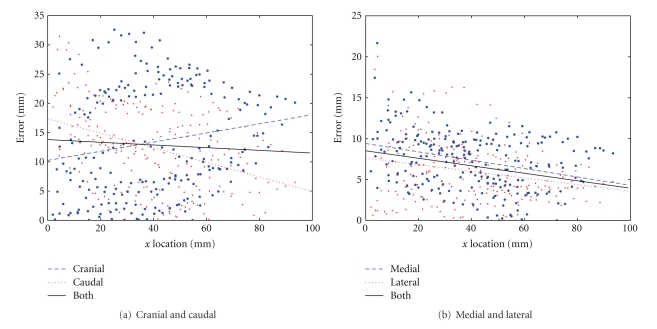
Skin estimation error versus *x* location.

To examine trends across all data sets, the absolute values of the skin estimation errors were plotted against *x*-axis location for all data sets (Figures 12(a) and 12(b)). These plots also show the best-fit linear regression for each individual direction, as well as both directions taken together. It should be noted that these best-fit lines are used only to illustrate trends and are not intended as a model of the data, as no linear relationship is expected to exist.

In both cranial-caudal and medial-lateral directions, the best-fit line for the combined data suggests a slight trend of increasing accuracy towards the chest wall. However, this may be partially a result of the nonelliptical shape of the breast at the chest wall as seen in Figure 10(a), leading to underestimation of the true error at these *x* locations. Similarly, the error at the nipple may be exaggerated due to the small coronal cross-section, where a slight misalignment can result in significant error. A more reasonable conclusion is that the error is approximately consistent along the *x*-axis of the breast.

Comparing Figures 12(a) and 12(b) confirms that the skin estimation technique is more accurate and consistent in the medial and lateral directions. This can be explained by the relative lack of data in the cranial-caudal direction due to the 45° angle between image planes. Since previous work assumed that the images were separated by 90°, it is likely that the current work is more accurate [10]. However, it is not possible to directly compare the two methods, as previous work did not assess errors relative to a ground truth.

Overall, the skin surface estimation was accurate to an average contour deviation of 13 mm in the cranial-caudal direction and 6 mm in the medial-lateral direction. This is considered acceptable given the number of assumptions required and the limited information available for estimating the 3D skin surface. As this is the first work to quantify the accuracy of a 3D skin surface estimation created from mammograms, it is difficult to determine whether this result is comparable to that of previous publications.

### 3.2. Internal Feature Estimation

**Figure 13:**
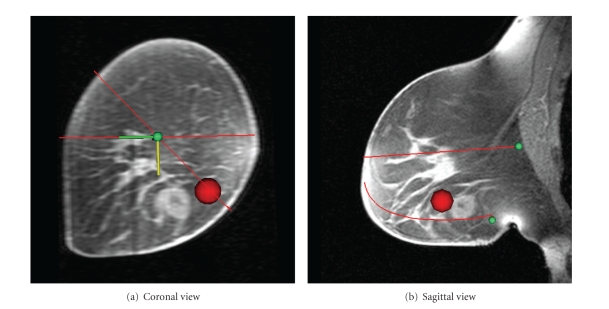
Comparison between feature reconstructed from mammograms and corresponding feature seen on MR.

Figure 13 shows a comparison between a reconstructed feature and the MR volume of the only data set containing a discrete feature visible in both modalities. The sphere marks the estimated region of the lesion resulting from backprojection, and the corresponding lesion on the MR is clearly visible as an opaque mass. The 3D Euclidean distance between the centroids of the true and estimated lesion location is approximately 12 mm. While it might seem reasonable to compare this value to the differences in Euclidean distance from lesion to nipple reported by Kita et al. in 2002, the two metrics are not in fact identical [10]. In the current work, Euclidean distance between lesion centroids accounts for absolute differences, whereas in Kita's work the two vectors may have different directions with a small difference in Euclidean distance. Similarly, this result cannot be compared to the error metric computed in the 2D mammographic space used by Behrenbruch et al. [11].

The radiology report for this patient states “mass lesion 4 o'clock right breast.” From visual inspection of Figure 13(a), the estimated 3D feature is indeed located at 4 o'clock. More precisely, the angle formed between the vectors from the ellipse centre to the two points (true and estimated lesion centroids) is 20°. This places the estimated lesion location within the same hour (30°) of the breast as the ground truth, providing a reasonably accurate 3D location of the lesion as seen on mammography. This metric is comparable to the “direction at front view” metric reported by Kita et al. and within a similar range of values [10].

The major difference between the methods described in this work and the methods of Kita et al. is the mapping of features into the prone acquisition geometry of MR imaging. As this similar to the geometry used for TSAR imaging, 3D ROI prediction from mammography will enable interpretation of TSAR images without reliance on MR data.

**Figure 14:**
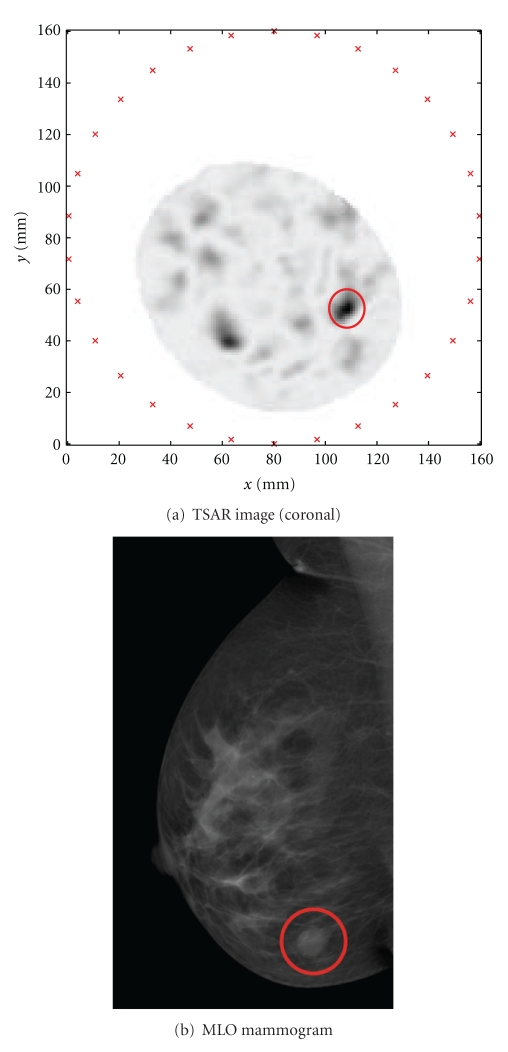
Comparison between experimental TSAR image and MLO mammogram with corresponding lesion encircled.

As a preliminary example, Figure 14 shows the experimentally acquired TSAR image and original MLO mammogram of this patient. Current TSAR protocol consists of immersing the breast in canola oil, then illuminating in a cylindrical pattern with a BAVA-D antenna described in [15]. At 200 antenna locations, measurement data are collected from 50 MHz to 15 GHz using a vector network analyzer. Concurrent to microwave data collection, a laser is used to obtain an accurate estimate of the skin location [16]. These data provide knowledge of the skin surface and are used to assist with processing the microwave data, which is then formed into an image using the delay-and-sum algorithm [17].

The 3D model of Figure 13(a) places the tumour at 4 o'clock, which is in agreement with the MR data and the radiologist's report. Even though Figure 13 was created in MR space instead of TSAR space, the identified ROI is likely to compare to the circled region of enhancement detected by TSAR, also located at 4 o'clock. However, it is difficult for a nonradiologist to identify the corresponding lesions circled in Figure 14.

Due to the immersion medium used in TSAR acquisition, the breast floats and changes shape relative to MR imaging, where the breast is hanging freely. This can be observed in Figures 13 and 14, which have very different shapes despite being images from the same patient. This illustrates the difficulties in comparing results from different modalities. In this example, it appears that the mammographic reconstruction is a closer match to the TSAR image than the MR data, but this is a coincidence resulting from a slight rotation of the breast between the MR and the TSAR images.

The data for the TSAR image shown in Figure 14(a) was acquired in a narrow range of slices around the anticipated tumour location. This resulted in an image that does not include the nipple or the chest wall landmarks. In the future, TSAR images will be used to remove the distortion from mammograms, but this was not possible in the current work as the landmarks are essential for matching breast area. Therefore, the method was developed and tested using MR images in place of TSAR, demonstrating the effectiveness of the technique.

### 3.3. Verification of Corresponding Features

**Figure 15:**
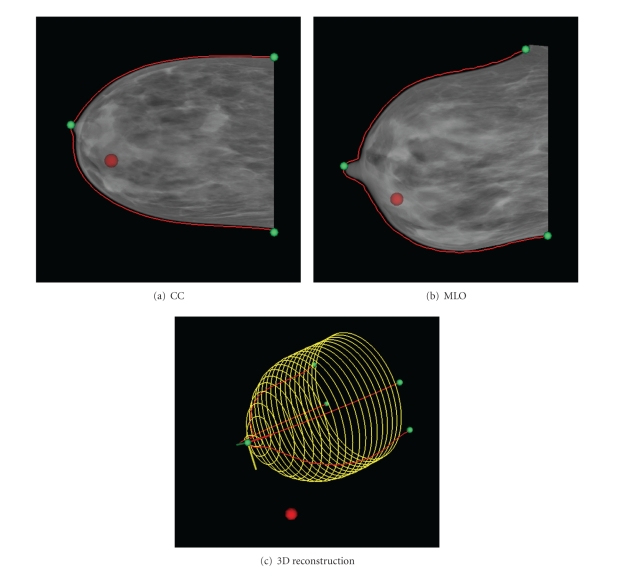
Identification of non-corresponding lesions results in mapping outside the breast volume.

A final application for the 3D estimation methods described in this paper is as a verification of features seen on mammography. Radiologists undergo extensive training in order to interpret medical images; without such experience, identification of the same feature on both mammographic views is difficult. However, once the ROI is reconstructed in 3D, it becomes obvious whether the two visible features correspond.  Figure 15 shows a data set where a suspected lesion is identified on the CC and MLO views. To the untrained eye, these locations could reasonably correspond, but the reconstruction (Figure 15(c)) clearly shows that the identified locations cannot exist within the breast. This verification has the potential to assist researchers and engineers in identifying true regions of interest.

## 4. Limitations

The comparison between MR ground truth and mammographic estimation is subject to errors related to the assumptions in aligning the two mammographic planes (see Section 2.2). This can be observed visually in Figure 10(b), where the MLO plane appears to be shifted relative to the true coronal cross-section. Such errors are not likely to impact the overall breast shape significantly, though improvements to the alignment would result in better error metrics.

In addition, each step of the method described in this work requires significant estimation and a large number of assumptions. As such, the technique has inherent inaccuracies and details that cannot be recovered with only two mammographic views, and the estimation should not be taken as an exact model of the 3D breast. However, for the intended purposes of assisting with microwave image interpretation, the accuracies obtained should suffice.

## 5. Conclusion

The skin surface and internal feature estimation techniques described in this paper have been found to be sufficiently accurate to assist with microwave image interpretation and provide a means of comparing TSAR results to the gold standard of mammography. While similar estimation techniques have been presented in the past, quantification of accuracy as compared to MR imaging is novel in the literature.

Future work will be to modify the mammogram undistortion technique to account for the geometric differences introduced by the TSAR immersion liquid. Following this, the 3D mammographic information will be incorporated into current TSAR image processing protocols.
